# Assessment of Genetic Diversity and Productive Traits in Crossbreed Cattle in the Caribbean Region, Colombia

**DOI:** 10.3390/genes16060677

**Published:** 2025-05-30

**Authors:** Andrés Rodríguez-Serrano, Marcos Ahumada-Velasco, Jesús María Cárdenas Beltrán

**Affiliations:** Profesional de Investigación, Universidad de Cartagena, Cartagena de Indias 130001, Colombia; camilo.rodriguez.serrano@gmail.com (A.R.-S.); jcardenas@unisalle.edu.co (J.M.C.B.)

**Keywords:** dual-purpose systems, genomic analysis, Gyr cattle, SNPs, homozygosity, inbreeding

## Abstract

Objectives: Evaluate the genetic diversity and productive traits of crossbred cattle in the Caribbean region of Colombia, through analyses derived from the assessment of the genome-wide single-nucleotide polymorphism (SNP). Methods: A total of 590 individuals and 66,098 SNPs were analyzed by principal components analysis (PCA) and detection of runs of homozygosity (ROH). The population was composed of 531 heifers marked as crossbreed and a group of 59 heifers marked as purebred Gyr. Additionally, allele frequencies were calculated for commercially important traits (*CSN2*, *CSN3*, *LGB*, *DGAT1*, *GH1*, *CAPN1_316*, *CAPN1_350*, *CAPN1_4751*, *CAST_282*, *CAST_2870*, and *CAST_2959*). Results: Global differences in PCA were 7.35%, and principal components explained 1.94% and 5.41% of the variation. Five ROH islands were identified in crossbred animals on chromosomes 2, 5, 7, 8, and 12. The majority of observed ROH classes were shorter than 2 Mb, 54% in crossbreed cattle and 47% in Gyr cattle. Individual inbreeding was 5.2% in crossbreed and 12% in Gyr cattle. Both groups had similar allelic and genotypic frequencies for most of the evaluated commercial traits. Only a wide variation was observed in the genes related to growth hormone (*GH1*) and Calpastatin *(CAST_2870* and *CAST_22959*). Crossbreed heifers had desired allele frequencies for better milk production and quality in the genes *CSN2*, *LGB*, *DGAT1*, and *GH1*, as well as in the genes *CAST_2870* and *CAST_2959*. Conclusions: Crossbreed cattle in the Colombian Caribbean region possess high genetic diversity and desirable allele frequencies to implement breeding and intense selection programs aimed at improving production yields.

## 1. Introduction

In biological terms, genetic diversity reflects the degree to which organisms can adapt to changing environmental conditions [1]. In livestock, it is the extent of selection processes that a population has undergone and the capacity to implement genetic improvement programs with the aim of increasing the frequency of desirable economic traits in animals [2]. Domestication and further selection of cattle have allowed the creation of a large number of breeds through factors such as mutations, migrations, and isolation [3]. These breeds have well-defined characteristics of interest to breeders, primarily dairy or meat production [4].

Due to this selection, and other practices such as intensive selective breeding, for a limited number of traits, intensification of production systems, controlled environmental conditions, and the globalization of breeding programs using common bulls, pure breeds have increasing inbreeding levels [2]. This increase in inbreeding could reduce the effective population size and result in inbreeding depression, probably leading to a decline in the expression of economically important traits [5]. For this reason, breeding programs and selection schemes must consider strategies to maximize genetic gain with minimal impact of inbreeding levels [6].

In contrast, crossbreed cattle in Colombia, due to the production system in which they develop, which are characterized by dual-purpose systems managed by low-income livestock farmers and a lack of technification, are often raised with minimal or no breeding programs, where production level is not the primary focus. Dairy production yield and average daily gains are about 3.5 L cow^−1^ and 420 g day^−1^, respectively. These animals compose 35% of the Colombian cattle herd [7], and despite their production levels, they represent nearly 50% of the national milk supply [8] and 40.5% of the total beef production chain [9].

Crossbreed cattle in dual-purpose systems of the Caribbean region of Colombia are primarily a product of crosses between different breeds, predominantly of the *Bos indicus* type; however, due to the lack of animal and productive records, their genealogical information is often partial or entirely unknown. It is common to have the indiscriminate use of breeders [10] and genetic diversity, inbreeding levels, and important economic traits are either unknown or under-evaluated. Consequently, implementing effective selection programs is difficult [11].

Some producers in the region incorporate pure breeds in their herds to increase productive performance and achieve heterosis. However, given the aforementioned challenges and lack of knowledge, not all crossbreeding efforts yield the expected advantages [10]. Faced with this situation, genomic analysis using single-nucleotide polymorphism (SNP) could be a tool to overcome the deficient genealogical information [12] and measure genetic diversity through methodologies like the detection of runs of homozygosity (ROH), which can identify identical homozygote genomic regions by descendance across the entire autosome genome and measure the coefficient of genomic inbreeding [13]. Additionally, SNP detection allows the identification of genomic regions associated with specific phenotypical parameters known as quantitative trait loci (QTL), among which are CSN2, CSN3, GH1, and LGB for milk production, and CAPN, CSN, and CAST for meat quality [14,15]. Therefore, the objective of this study was to evaluate the genetic diversity and productive traits of crossbred cattle in the Caribbean region of Colombia, through analyses derived from the assessment of the genome-wide single-nucleotide polymorphism (SNP), and through this analysis, test the hypothesis that the management of crossbred cattle in dual-purpose systems in the region leads to reduced genetic diversity and a lower prevalence of commercially productive traits in the genome.

## 2. Materials and Methods

The study was conducted in the Caribbean Region of Colombia, and hair follicle samples were collected from 590 heifers from Bolivar n = 75, Cordoba n = 506, and Sucre n = 6. Heifers were selected in order to make a descriptive and exploratory analysis of the genetic diversity and prevalence of commercially productive traits in the genome, which works as a tool for reproduction and genetic improvement programs. Heifers were selected from small-scale producers, body weights ranging between 300–350 kg and body condition scores between 5–8 (on a 1–10 scale). Sampling was conducted during the mandatory cattle vaccination campaigns organized by the Colombian government, during which each producer confined the herd on their own properties. The confinement was conducted only once and lasted no more than 20 min. Tail hair removal was performed with a quick and instantaneous upward movement, extracting at least 20 hairs with intact follicles stored in paper envelopes. Initially, the plan was to sample crossbred heifers, but the cattle population of the producers who participated in the sampling included a significant proportion of pure Gyr breed animals, which are often used as genetic improvement material. For this reason, it was decided to differentiate the two groups—crossbred and pure Gyr—to conduct the analyses.

DNA extraction was performed using the Termofisher, MagMAX™ CORE Nucleic Acid Purification kit, Thermo Fisher Scientific, Waltham, MA, USA. Genotyping and commercially important genes identifications were performed using the Termofisher’s software, Axiom™ V. 4.0, Bovine Genotyping 100 K. Data was pruned by quality control using software PLINK 1.9 [16] with a call rate (number of markers successfully identified) ≥ 95%, minor allele frequency (MAF) < 0.05, Hardy–Weinberg equilibrium > 0.000001, and missing genotype < 10%. After quality control, 66,098 SNPs were preserved, and no heifers were removed. The population was composed of 531 heifers marked as crossbreed and a group of 59 heifers marked as pure breed Gyr.

### 2.1. Genomic Analysis

A principal component analysis (PCA) was performed, using the genetic distances between individuals, calculated with the command “--distance-matrix” from PLINK 1.9 software. Additionally, to measure the inbreeding coefficient, a runs of homozygosity analysis (FROH) was performed using the RSdtudio V.4.4.1 package “DetectRuns” [17] with the “SlidingRuns” method, using the following parameters: (a) Window size 50 SNP; (b) minimum ROH length 1000 bps; (c) Maximum gap per ROH 10^6^; and (d) Minimum SNP 20. The FROH was estimated following the method described by [18].

$$F_{ROH}\frac{L_{ROH}}{L_{AUT}}$$

where LROH is the total length of all ROH detected in the individual’s autosomes, and LAUT is the entire length of the autosomal genome. Additionally, the population was categorized into five length classes (1–2 Mb, 2–4 Mb, 4–8 Mb, 8–16 Mb, and >16 Mb) to analyze the FROH distribution. The percentage of SNPs existing within a ROH was calculated, and potential hotspots or ROH in the genome were identified when the percentage exceeded a 50% threshold [19]. SNPs found within a ROH were explored for annotated genes on the *Bos taurus* assembly ASR-UCD1.2 in the genomic data viewer available at https://www.ensembl.org/Bos_taurus/Info/Index, accessed on 15 February 2025.

### 2.2. Commercially Important Traits

Genotypic frequencies for commercially important traits (CSN2, CSN3, LGB, DGAT1, GH1, CAPN1_316, CAPN1_350, CAPN1_4751, CAST_282, CAST_2870, and CAST_2959) were calculated using the Hard–Weinberg equation *(p*^2^ *+* 2*pq + q*^2^*)*, where *p*^2^ is the frequency of the dominant homozygous dominant genotype, 2*pq* is the frequency of the heterozygous genotype, and *q*^2^ is the frequency of the homozygous recessive genotype. Allele frequencies were calculated from genotypic frequencies, and the proportion of individuals that have important genes for milk and beef production was established.

## 3. Results

**Figure 1:**
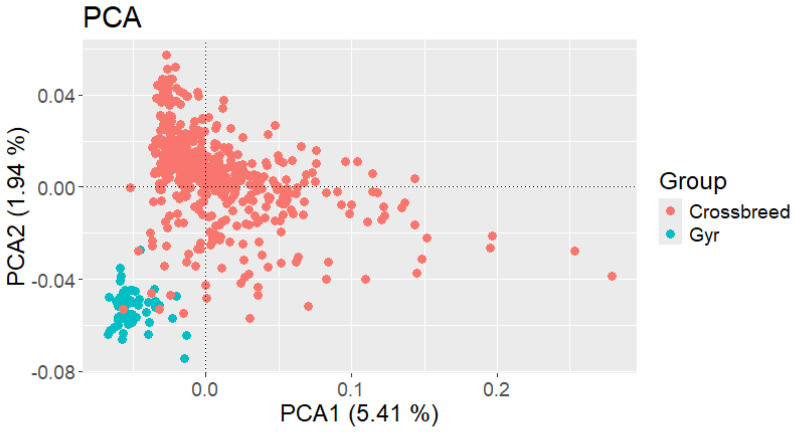
Population groups according to principal component analysis (PCA).

According to the PCA, the two evaluated groups (Crossbreed and Gyr cattle) exhibit molecular differences. However, they are closely related, primarily because crossbreed cattle in the region consist of *Bos indicus* breeds, and Gyr cattle have an important presence in the Caribbean region of Colombia. Notably, some of the farmer’s heifers registered ass crossbred show a closer relationship to the Gyr population; this observation underscores the lack of accurate records, which is a common situation in small livestock farmers. Global differences in PCA were 7.35%, and principal components explained 1.94 % and 5.41% of the variation (Figure 1). When the origin of the crossbred heifers (farm) was included in the PCA analysis to assess whether small-scale producers utilized different breeds in their farms according to specific production goals, no differences were detected, probably because the crossbreed cattle across the evaluated population were composed of similar breeds.

**Figure 2:**
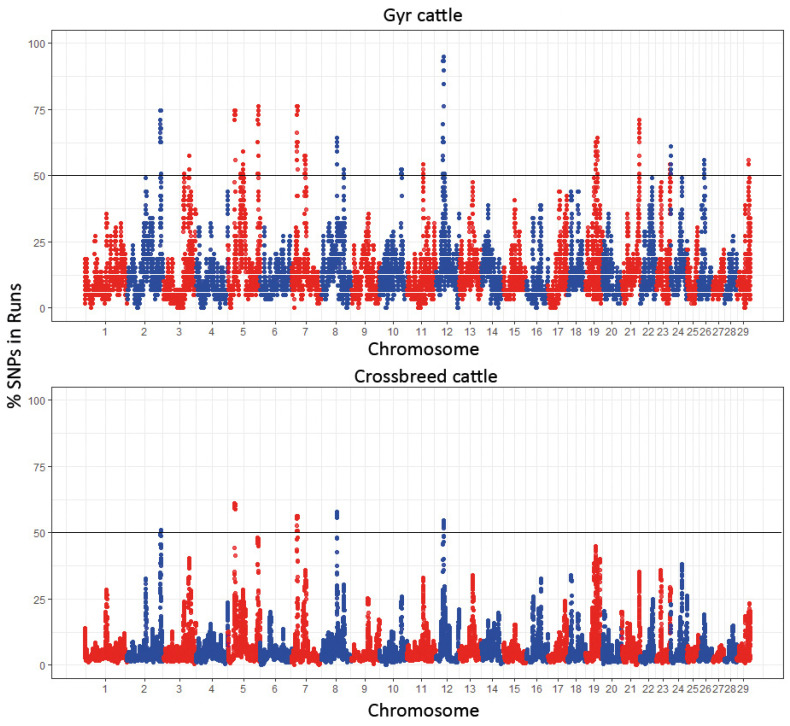
SNP frequency within run of homozygosity in the autosome of crossbreed and Gyr cattle in the Caribbean region of Colombia.

Consequently, with a widely dispersed genomic population, crossbred cattle had fewer ROH islands compared to the Gyr population, as illustrated in the Manhattan plot (Figure 2). Using the 50% threshold, five ROH islands were identified in crossbred heifers on BTA 2, 5, 7, 8, and 12, whereas the Gyr cattle showed 31 ROH islands distributed across BTA 2, 3, 5, 7, 8, 10, 11, 12, 19, 21, 23, 24, 26, and 29.

ROH islands were found because the SNPs are shared by more than 50% of crossbred heifers, serving as evidence of selection. These islands revealed 60 annotated genes (Table 1).

The majority of observed ROH classes were shorter than 2 Mb, 54% in crossbreed cattle and 47% in Gyr cattle (Table 2). The maximum number of ROH observed per cow was 105 in crossbreed cattle and 114 in Gyr cattle, while the minimum ROH for the same groups was 3 and 58, respectively.

**Figure 3:**
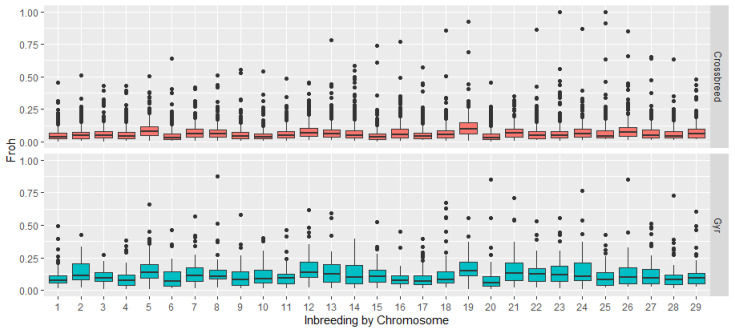
Inbreeding based on ROH among the autosome of crossbreed and Gyr cattle from the Colombian Caribbean region.

Mean inbreeding values are low in both groups; individual inbreeding rates were 5.2% in crossbreed and 12% in Gyr cattle. Mean FROHCrh across the autosome was low, especially in crossbreed cattle, compared with the Gyr population, 7% and 12%, respectively; the highest mean values were found for BTA 5, 12, and 19, in both groups (Figure 3). These indicate that even in the pure breed, genetic diversity is high; these values are lower in the crossbreed group and similar in the pure breed group.

Both groups have similar allelic and genotypic frequencies for most of the evaluated commercial traits; only a wide variation was observed in the genes related to growth hormone (*GH1*) and Calpastatin *(CAST_2870* and *CAST_22959*) (Table 3). Homozygote variants and desired alleles were less frequent in the evaluated population for genes of commercial importance in meat production, probably because crossbreed cattle in dual-purpose systems in the Colombian Caribbean region are not selected and raised for high average daily gains, and breeding with *Bos taurus* breeds specially for meat production is uncommon.

## 4. Discussion

The PCA results showed a clear differentiation between the two evaluated groups. This is evidence that small-scale producers try to maintain their purebred population, either as a personal preference or because Gyr cattle are a locally adapted breed that significantly improves farm milk yields. When only crossbred heifers were analyzed, no genetic differences were observed. These results are consistent with those reported by Toro-Ospina et al. [20], who found no genetic differentiation among Caqueteño Cattle (a local breed from Colombia) from different farms. Rosero et al. [10] registered that crossbreed cattle in dual-purpose systems in Colombia did not exhibit genetic separation compared with crossbreed individuals with a clear phenotype of *Bos indicus* or *Bos taurus* animals, which leads to the use of control breeds to define better the groups that constitute multiracial individuals; when these authors incorporated pure breeds in their genomic analysis, they observed that crossbreed cattle of Piedemonte Llanero-Colombia have predominance of Brahman, Gyr, and Holstein breeds.

The PCA results in the present study showed greater dispersion among crossbred individuals compared to the Gyr population. Indicating higher genetic variability, this outcome is expected because, historically, small-scale producers in dual-purpose systems of the Colombian Caribbean region, among their herds that have a high presence of various breeds of *Bos indicus* animals, can eventually include animals with greater *Bos taurus* influence, either as crossbred or purebred individuals. These results are similar to those reported by Naranjo et al. [21] in crossbreed cattle for milk production from various regions of Colombia, which exhibited greater genetic dispersion compared with pure breeds. In addition, the study also compared the genomic information of Brahman, Gyr, Brown Swiss, Holstein, Romosinuano, Jersey, and Senepol breeds, highlighting that crossbreed animals are in the center of these groups, revealing a multiplicity of lineages used for crossbreeding, with an important component of Brahman and Gyr breeds.

The wide genomic dispersion observed in crossbred cattle within dual-purpose systems in Colombia, through different breeds of *Bos indicus* and *Bos taurus* type, suggests a historically indiscriminate use of breeds in the absence of a breeding program [22].

The genes with an associated QTL were as follows: *AHDC1*, which could be related to body weight, energy metabolism, and clinical and subclinical ketosis [23,24]; *TENT5B*, involved in innate immune response [25]; *GLYCAM1*, which have an important role in regulating milk protein [26]; *PEDE1B*, identified as important to growth and carcass quality [27]; *GTSF1*, involved in reproduction processes through its role in the Argonaute proteins activity [28]; *CALCOCO1*, a gene candidate for meat quality in Angus cattle through cellular phosphate and glucose metabolism, as well as protein synthesis and degradation [29,30]; *ATP5MC2*, related with oxidative phosphorylation [31]; *MOB3A* and *PIGO* genes, which could be associated with weight gain and meat quality [32,33]; *STARD13*, a candidate gene for average daily gain (ADG) in cattle [34]; and *PDS5B*, a candidate gene for hematological parameters in Holstein [35]. No commercially important traits were observed in the ROH islands founded for the crossbreed heifer population evaluated, probably because very high production yields are not the principal selection criteria for small-scale producers of the region. Normally for them, it is more important to have resistant and adapted animals that can live and produce in an environment that does not always provide the resources for higher production levels or maintain specialized cattle.

In creole breeds raised in similar environmental conditions, it had been reported similar proportions of ROH length classes shorter than 2 Mb, 62.82% in the Caqueteño breed, and 76.0% in the Tropical milking Criollo breed [20,36]. In Gyr populations, Peripolli et al. [37] registered a proportion of 59.42% in the length class <2 Mb, indicating ancient inbreeding in both groups despite the lack of breeding programs.

Inbreeding values were similar to those registered by Martinez-Rocha et al. [36], in the Tropical milking Criollo, who reported a mean FROHCrh of 11.26%, and mean values across the autosome between 0 and 20%. FROH inbreeding can be higher in specialized breeds due to the high rate of selection to which they are subjected. Lozada-Soto et al. [38] reported a FROH between 15% and 17% in the Brown Swiss and Jersey Cattle, respectively, and Betancur-Zambrano et al. [39] reported a FROH of 28% in the Colombian Holstein cattle.

In dual-purpose cattle, the presence of desired commercial traits for both dairy and meat production is important. Among the genes of commercial interest for dairy production is Kappa casein (*CSN3*), where the BB allele is reported to improve cheese yield [40]. In the evaluated heifers, this allele is the least frequent, particularly in its homozygote form.

For Beta casein (*CSN2*), the A2A2 allele has been associated with enhanced production of essential amino acids and health benefits for humans [41], whereas the A1A1 allele is linked to the best milk quality for cheese production [42]. In the present study, the evaluated individuals showed high frequencies of the A2A2 allele, which is important in small-scale producers, where part of the milk produced is designated for self-consumption.

Regarding the Beta lactoglobulin (*LGB*), the presence of the A allele is associated with the best milk protein concentration (ideal for cheese making), whereas genotype BB is associated with better fats and casein content as well as high milk production [43]. In the evaluated population, the B allele was present at a high frequency in both groups.

For the CoA:diacylglycerol acyltransferase 1 (*DGAT1*), the AA variant codes for K lysine, which is associated with improved production of milk fat and protein [44]. The Gyr population showed a higher frequency of the AA variant compared to the crossbreed group. A similar trend was observed for the Growth hormone (*GH1*) gene, where the G allele has been reported as beneficial to milk composition and production [45], and Gyr group had a higher frequency of the GG variant compared to the crossbreed individuals.

In Calpain Genes (*CAPN1_316*, *CAPN1_530*, and *CAPN1_4751*), the desired alleles due to their relationship with meat tenderness are C, A, and C, respectively [46,47]. In the evaluated population, both crossbreed and Gyr cattle showed minimal frequencies of these alleles or, in some cases, their complete absence. Regarding the Calpastatin genes (*CAST_282*, *CAST_2870*, and *CAST_2959*), the desired alleles are C, G, and A, respectively [48], which in the evaluated population have frequencies close to 50%.

## 5. Conclusions

The evaluated population of crossbreed cattle in dual-purpose systems of the Colombian Caribbean Region showed a wide genomic dispersion, indicating high genetic diversity. No genomic differentiation was observed among individuals within the crossbreed group, probably because the evaluated crossbreed heifers are composed of similar breeds with a strong *Bos indicus* genetic component.

Despite a lack of accurate records and the absence of a breeding program, homozygous regions are short, and the inbreeding coefficient based on ROH is low even in heifers recorded as pure Gyr. A total of five ROH islands were detected; however, no genes with associated common QTLs were identified, indicating that probably the population has not been subjected to intense selection processes aimed at achieving high production yields in either milk or weight gains.

Nevertheless, crossbreed heifers have the desired allele frequencies for better milk production and quality in the genes *CSN2*, *LGB*, *DGAT1*, and *GH1*, as well as in the genes *CAST_2870* and *CAST_2959*, which are important for meat production and tenderness.

Crossbreed cattle in the Colombian Caribbean region possess high genetic diversity and desirable allele frequencies to implement breeding and intense selection programs aimed at improving production yields. However, this should be in parallel with proper records management, improved feed and water supply, and the maintenance of optimal sanitary conditions to enhance gene expression for either milk or meat production.

## Figures and Tables

**Table 1:** Description of the ROH islands detected in the autosomal genome of the crossbreed cattle population in the Colombian Caribbean Region.

BTA	nSNP	From	To	Associated Gene
2	22	125,691,492	126,383,381	*FGR*, *AHDC1*, *WASF2*, *MAP3K6*, *TMEM222*, *SYYL1*, *GPN2*, *KDF1*, *TENT5B*
5	52	25,259,925	26,964,045	*GLYCAM1*, *PDE1B*, *GTSF1*, *COPZ1*, *MAP3K12*, *TF7*, *CALCOCO1*, *HOXC4-8-10-11-13*, *SMUG1*, *ATP5MC2*
7	51	19,764,739	21,612,020	*FSD1*, *NMRK2*, *ATCAY*, *DAPK3*, *TEKTIP1*, *GIPC3*, *SHF ANKRD24*, *SHD*, *TMIGD2*, *MATK*, *CELF5*, *NCLN*, *TLE6*, *MOB3A*, *S1PR4*, *APBA3*
8	51	58,010,694	59,902,102	*RHF32*, *SPATA31G1*, *PIGO*, *STOML2*, *ATOSB*, *RUSC2*, *CIMIP2B*, *TESK1*, *CD72*, *SIT1*
12	48	27,933,503	29,563,846	*STARD13*, *KL*, *PDS5B*, *N4BP2L2*, *ZAR1L*, *FRY*, *RXFP2*

**Table 2:** Number (nROH) and mean length (in Mb) of the ROH islands in different length classes.

Group		<2	2–4	4–8	8–16	>16
Crossbreed	n ROH	14,290	9399	2028	663	181
	Mean length	1.45	2.64	5.4	10.85	23.5
Gyr	n ROH	2493	1860	529	236	142
	Mean length	1.44	2.68	5.42	11.19	26.11

**Table 3:** Genomic and allelic frequencies for commercial traits in crossbreed and Gyr cattle from the Colombian Caribbean region.

GEN	SNP ID	Description	Genotypic Frequencies						Allelic Frequencies			
			Gyr	Cross	Gyr	Cross	Gyr	Cross	Gyr	Cross	Gyr	Cross
			**A1A1**		**A1A2**		**A2A2**		**A1**		**A2**	
*CSN2*	rs43703013	Beta Casein	0.00	0.00	0.17	0.08	0.83	0.92	0.08	0.04	0.92	0.96
	rs43703011		0.02	0.01	0.19	0.17	0.8	0.82	0.11	0.09	0.89	0.91
			**AA**		**AB**		**BB**		**A**		**B**	
*CSN3*	rs43706475	Kappa Casein	1.00	1.00	0.00	0.00	0.00	0.00	1.00	1.00	0.00	0.00
	rs110014544		0.78	0.80	0.22	0.19	0.00	0.01	0.89	0.11	0.89	0.11
*LGB*	rs110180463	Beta lactoglobulin	0.00	0.01	0.02	0.17	0.98	0.9	0.01	0.10	0.99	0.82
	rs110066229		0.19	0.11	0.46	0.48	0.36	0.41	0.42	0.35	0.58	0.65
	rs110641366		0.00	0.06	0.10	0.42	0.90	0.51	0.05	0.27	0.95	0.73
	rs109625649		0.17	0.10	0.47	0.48	0.36	0.42	0.41	0.34	0.59	0.66
		CoA:diacylglycerol acyltransferase 1	**AA**		**AG**		**GG**		**A**		**G**	
*DGAT1*	rs109234250		0.84	0.65	0.14	0.31	0.02	0.04	0.91	0.81	0.09	0.19
		Growth hormone	**TT**		**TG**		**GG**		**T**		**G**	
*GH1*	rs109191047		0.00	0.50	0.20	0.35	0.98	0.60	0.01	0.22	0.99	0.78
*CAPN1_316*	rs17872000	Calpain	**CC**		**CG**		**GG**		**C**		**G**	
			0.00	0.00	0.00	0.10	1.00	0.90	0.00	0.05	1.00	0.95
*CAPN1_530*	rs17871051		**AA**		**AG**		**GG**		**A**		**G**	
			0.00	0.00	0.07	0.12	0.93	0.88	0.03	0.06	0.97	0.94
*CAPN1_4571*	rs17872050		**TT**		**TC**		**CC**		**T**		**G**	
			0.73	0.69	0.27	0.27	0.00	0.03	0.86	0.83	0.14	0.17
*CAST_282*	rs110955059	Calpastatin	**CC**		**CG**		**GG**		**C**		**G**	
			0.29	0.26	0.42	0.53	0.29	0.22	0.50	0.52	0.50	0.48
			**AA**		**AG**		**GG**		**A**		**G**	
*CAST_22870*	rs41255587		0.05	0.19	0.27	0.51	0.58	0.30	0.19	0.45	0.81	0.55
*CAST_22959*	rs109221039		0.69	0.39	0.29	0.48	0.02	0.13	0.84	0.63	0.16	0.37

## Data Availability

The data presented in this study are available on request from the corresponding author due to confidentiality.
